# Significant Role of Collagen XVII And Integrin β4 in Migration and Invasion of The Less Aggressive Squamous Cell Carcinoma Cells

**DOI:** 10.1038/srep45057

**Published:** 2017-03-22

**Authors:** Jyri M. Moilanen, Stefanie Löffek, Nina Kokkonen, Sirpa Salo, Juha P. Väyrynen, Tiina Hurskainen, Aki Manninen, Pilvi Riihilä, Ritva Heljasvaara, Claus-Werner Franzke, Veli-Matti Kähäri, Tuula Salo, Markus J. Mäkinen, Kaisa Tasanen

**Affiliations:** 1 Department of Dermatology, PEDEGO Research Unit, Oulu Center for Cell-Matrix Research, MRC Oulu, University of Oulu and Oulu University Hospital, Finland; 2 Skin Cancer Unit of the Dermatology Department, Medical Faculty, West German Cancer Center, University of Duisburg-Essen, Germany; 3 Oulu Center for Cell-Matrix Research, Biocenter Oulu and Faculty of Biochemistry and Molecular Medicine, University of Oulu, Oulu, Finland; 4 Department of Pathology, Research Unit of Cancer and Translational Medicine, MRC Oulu, University of Oulu and Oulu University Hospital, Oulu, Finland; 5 Department of Dermatology, Turku University Hospital, MediCity Research Laboratory, University of Turki, Turku, Finland; 6 Department of Dermatology, Medical Center, University of Freiburg, Freiburg, Germany; 7 Research Unit of Cancer and Translational Medicine, MRC Oulu, University of Oulu and Oulu University Hospital, Finland; 8 Department of Oral and Maxillo-facial Diseases, University of Helsinki, Finland; 9 HUSLAB, Department of Pathology, Helsinki University Central Hospital, Finland; 10 Department of Oral Diagnosis, Oral Pathology Division, Piracicaba Dental School, University of Campinas, Piracicaba, São Paulo, SP-13414-903, Brazil

## Abstract

Collagen XVII and integrin α6β4 have well-established roles as epithelial adhesion molecules. Their binding partner laminin 332 as well as integrin α6β4 are largely recognized to promote invasion and metastasis in various cancers, and collagen XVII is essential for the survival of colon and lung cancer stem cells. We have studied the expression of laminin γ2, collagen XVII and integrin β4 in tissue microarray samples of squamous cell carcinoma (SCC) and its precursors, actinic keratosis and Bowen’s disease. The expression of laminin γ2 was highest in SCC samples, whereas the expression of collagen XVII and integrin β4 varied greatly in SCC and its precursors. Collagen XVII and integrin β4 were also expressed in SCC cell lines. Virus-mediated RNAi knockdown of collagen XVII and integrin β4 reduced the migration of less aggressive SCC-25 cells in horizontal scratch wound healing assay. Additionally, in a 3D organotypic myoma invasion assay the loss of collagen XVII or integrin β4 suppressed equally the migration and invasion of SCC-25 cells whereas there was no effect on the most aggressive HSC-3 cells. Variable expression patterns and results in migration and invasion assays suggest that collagen XVII and integrin β4 contribute to SCC tumorigenesis.

Cutaneous squamous cell carcinoma (SCC) is among the most common carcinomas and its incidence has been rising rapidly over the past two decades^1^. In the process of progression to invasive tumor SCC cells invade the basement membrane of dermo-epidermal junction^2^. Hemidesmosomes (HD) are multiprotein focal adhesion complexes that attach epithelial cells strongly to the underlying basement membrane^2^. Loss of attachment via disassembly of HDs is crucial for SCC cells to migrate and invade^3^,^4^.

HDs consist of α6β4 integrin, collagen XVII (BP180), BP230, plectin and tetraspanin CD151^2^. The binding of HDs to underlying basement membrane is mediated by interactions of α6β4 integrin and collagen XVII with laminin 332, which is the major component of anchoring filaments^2^. The roles of HD components and their binding partners in SCC carcinogenesis has been studied widely, and the importance of laminin 332 and α6β4 integrin in SCC cell migration and invasion is well established^5^,^6^,^7^,^8^,^9^,^10^,^11^. Laminin 332 is thought to be crucial for the invasion of SCC cells and it promotes their migration as both a soluble factor and an insoluble substrate^7^. Especially, the γ2 chain of laminin 332 is overexpressed at the invasive front of the SCC tumors and frequently expressed as a monomer in SCC and other malignant tumours^7^,^8^,^9^. α6β4 integrin is often upregulated in carcinoma cells. Moreover, there is strong evidence that it facilitates the formation of some carcinomas as well as the migration, invasion, and survival of carcinoma cells^6^,^10^,^11^. Both laminin 332 and α6β4 integrin are shown to be required for tumorigenesis in a murine xenograft model of human SCC^12^. Collagen XVII has a well-established function in keratinocyte adhesion and migration^13^,^14^,^15^, it is critical for the maintenance of hair follicle stem cells^16^ and it is abnormally distributed and up-regulated in actinic keratosis, Bowen’s disease, basal cell carcinomas and especially in the invasive areas of cutaneous and mucosal SCCs growth^17^,^18^,^19^,^20^.

Recent studies have revealed that the expression of collagen XVII is essential for the survival and function of cancer stem cells in colon and lung cancer^21^,^22^. These findings and the involvement of laminin 332 and integrin α6β4 for the pathogenesis of SCC and other malignancies led to us to hypothesize that collagen XVII may also have a function in migration and invasion of SCC cells. To clarify the relationship between these three cutaneous adhesion proteins in SCC carcinogenesis we first analyzed simultaneously the expression of collagen XVII, laminin γ2 and integrin β4 in human samples cutaneous SCC and its precursors, actinic keratosis and Bowen’s disease as well as chemically induced skin carcinomas of mice. Another focus of our work was to assess and compare the function of hemidesmosomal binding partners, collagen XVII and integrin β4, in SCC cells using viral knockdown of collagen XVII and integrin β4. Our study demonstrates a clear disturbance in migration and invasion in collagen XVII- and integrin β4-deficient SCC cells.

## Results

### Increased expression and intensity variation of collagen XVII, laminin γ2 and integrin β4 in cutaneous squamous cell carcinoma and its precursors, actinic keratosis and Bowen’s disease

**Figure 1:**
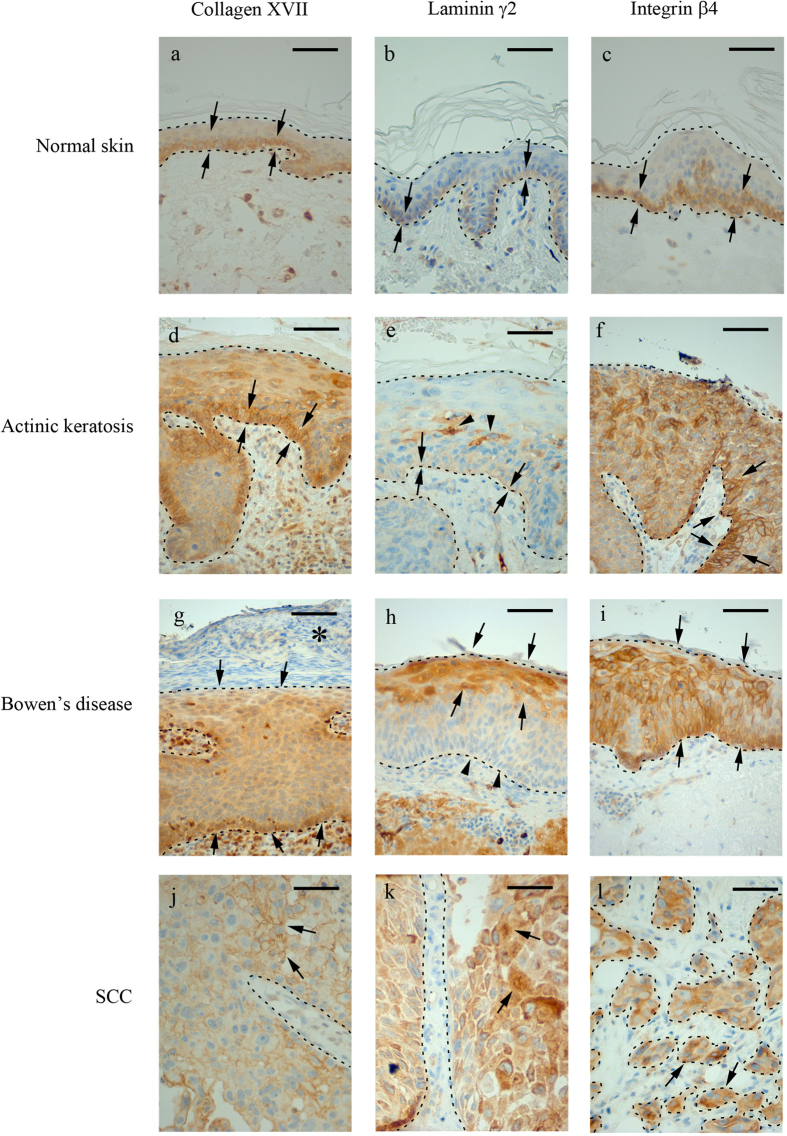
The expression of collagen XVII, integrin β4 and laminin γ2 in tissue microarray samples of actinic keratosis, Bowen’s disease and squamous cell carcinoma. In normal skin collagen XVII (**a**) and integrin β4 (**c**) are observed in basal cells of the epidermis (arrows), and laminin γ2 (**b**) shows weak immunoreaction at the basement membrane area (arrows). In actinic keratosis collagen XVII (**d**) and integrin β4 (**f**) immunoreaction is observed in both basal and supra basal cells with varying intensity (arrows). Only keratinous layer remains negative. Laminin γ2 (**e**) is preserved at the basement membrane area (arrows), but aberrant immunoreaction is observed in individual supra basal cells (arrow heads). In Bowen’s disease collagen XVII (**g**) and integrin β4 (**i**) immunoreaction are observed in all epidermal cell layers (arrows), but the parakeratotic plaque (asterisk) remains negative (**g**). Laminin γ2 (**h**) shows positivity in the upper half of the cells (arrows) while the basement membrane area is indistinct (arrow heads). Squamous cell carcinoma shows aberrant immunoreaction against collagen XVII, laminin γ2 and integrin β4. In collagen XVII (**j**), immunoreaction is predominantly membranous (arrows), in laminin γ2 (**k**) cytoplasmic (arrows), and integrin β4 (**l**) shows mostly cytoplasmic immunoreaction (arrows), as membranous immunoreaction is weaker than in normal and dysplastic cells. Epithelial areas are marked with dotted line. Original magnification 200x. Scale bar 100 μm.

**Figure 2:**
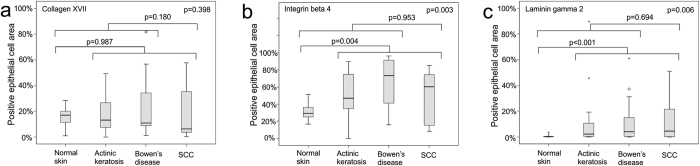
Boxplots displaying the expression of collagen XVII, integrin β4 and laminin γ2 in normal skin, actinic keratosis, Bowen’s disease, and squamous cell carcinoma. The proportion of positive immunoreaction in epithelial cells relative to total epithelial cell area was calculated from tissue microarray (TMA) samples of normal skin (n = 13), actinic keratosis (n = 36), Bowen’s disease (n = 30) and squamous cell carcinoma (n = 16). *p* Values are for Kruskall-Wallis test or Mann-Whitney U test as appropriate.

Immunostaining of human cutaneous SCC samples demonstrated high expression of laminin γ2, collagen XVII and integrin β4, especially in basal hyperplastic cells, but also in individual invasive cells (Fig. 1). The staining pattern of collagen XVII and integrin β4 were very similar. For quantitative analysis of patient samples, we calculated the proportion of positive immunoreaction in epithelial cells relative to total epithelial cell area in tissue microarray (TMA) samples of normal skin, actinic keratosis, Bowen’s disease and SCC (Fig. 1). The proportion of cells positive for laminin γ2 (*p* < 0.001) and integrin β4 (*p* = 0.004) was increased in SCC, actinic keratosis, and Bowen’s disease relative to normal skin (Fig. 2). For integrin β4 the highest proportion of positive epithelial/tumor cells were found in Bowen’s disease samples and for laminin γ2 in SCC samples (Fig. 2). Regarding immunopositivity for collagen XVII difference between groups was not detected (*p* = 0.398), since the amount of cells positive for collagen XVII showed higher variation in samples of actinic keratosis, Bowen’s disease and SCC than in normal skin (Fig. 2). Especially many cases of SCC showed markedly increased expression of collagen XVII, while others were totally negative. In the SCC samples, Spearman rank correlation coefficients (ρ) indicated that the proportion of positive epithelial cells for different markers showed some correlation (collagen XVII and integrin β4: ρ = 0.336; collagen XVII and laminin γ2: ρ = 0.609; integrin β4 and laminin γ2: ρ = 0.310).

### Collagen XVII and laminin γ2 are up-regulated in chemically induced skin carcinomas of mice

Immunohistochemical staining of 7,12-dimethylbenz(α)anthracene (DMBA) and 12-O-tetradecanoylphrobol-13-acetate (TPA) -induced skin carcinomas of mice was performed to compare the expression patterns of collagen XVII and laminin γ2 during *de novo* carcinogenesis. The two-stage protocol of skin carcinogenesis results in the outgrowth of highly differentiated benign papilloma that may progress to malignant SCC and thus models human skin SCC initiation, promotion and progression^23^. Immunostaining showed that the expression of collagen XVII and laminin γ2 chain were up-regulated in basal hyperplastic tissue of benign papilloma (Fig. S1a,c) and in the individual invasive cells of malignant SCCs (Fig. S1b,d). Both collagen XVII and the laminin γ2 chain signals in the basement membrane zone were also much stronger in all papilloma and carcinoma samples compared to the normal skin sample (Fig. S1).

### Increased expression of collagen XVII, laminin γ2 and integrin β4 in SCC cells

**Figure 3:**
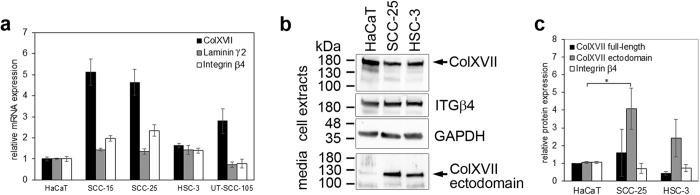
The expression of collagen XVII and integrin β4 in squamous cell carcinoma cells. (**a**) Total RNA was isolated and mRNA levels were measured by quantitative RT-PCR analysis. The expression of collagen XVII, laminin γ2 chain and integrin β4 is increased in SCC-15, SCC-25 and HSC-3 cells when compared to HaCaT keratinocytes. (**b**) Immunoblotting of cell extracts and media with the polyclonal human NC16A and integrin β4 antibodies shows that collagen XVII and integrin β4 proteins are expressed in SCC-25 and HSC-3 cells and that the shed ectodomain of collagen XVII (120 kDa) is present in media samples. (**c**) The amount of full-length collagen XVII and integrin β4 in cell extract samples, and the shed ectodomain of collagen XVII in cell culture media samples was quantified using the ImageJ software. *p* Values are for independent sample *t*-test, *p < 0.05.

To analyze whether the expression of HD components are increased in SCC cell lines we determined the relative expression levels of collagen XVII, laminin γ2 and integrin β4 in less (SCC-15 and SCC-25) and more aggressive (HSC-3) oral mucosal cell lines^24^,^25^ and in cutaneous UT-SCC-105 cells^26^ by qRT-PCR. Compared to HaCaT keratinocytes collagen XVII, laminin γ2 chain and integrin β4 were overexpressed in all SCC cells except UT-SCC-105 cells (Fig. 3a). Collagen XVII expression was highest (5.13 fold) in SCC-15 cells and also elevated in other SCC cells. SCC-15 and SCC-25 cells had also elevated laminin γ2 (1.36–1.43 fold increase) and integrin β4 (1.40–2.35 fold increase) expression. Western blot analysis revealed that the amount of the full-length collagen XVII was higher in SCC-25 cell extracts than in HaCat extracts, and lower in HSC-3 cell samples, but these differences were not statistically significant (Fig. 3b,c). The amount of shed ectodomain in the cell culture media samples of SCC-25 was significantly higher compared to HaCat cells (*p* = 0.042) and also increased, although not significantly, in HSC-3 cells (*p* = 0.246) (Fig. 3b,c).

### Knockdown of collagen XVII and integrin β4 inhibits the migration and invasion of SCC-25 cells *in vitro*

**Figure 4:**
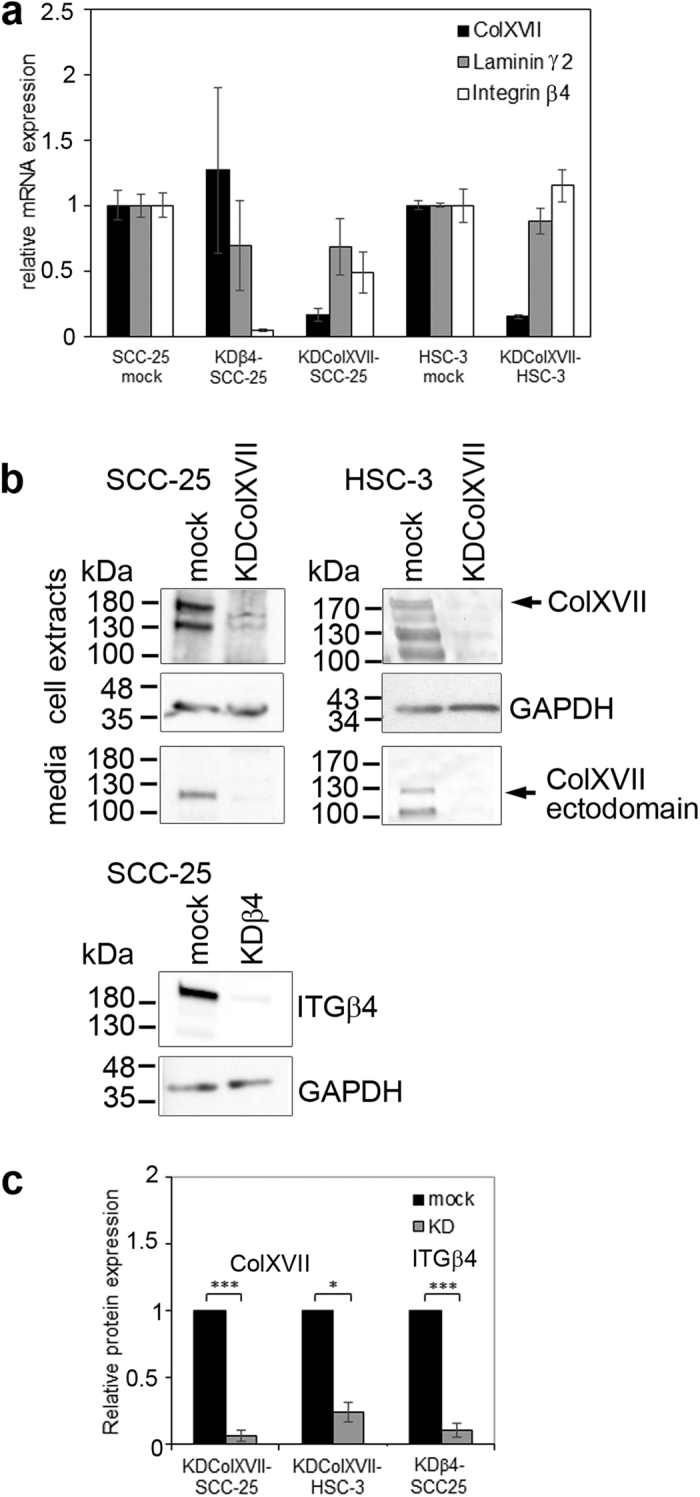
The knockdown of collagen XVII and integrin β4 in squamous cell carcinoma cells. (**a**) SCC cell lines with knockdown (KD) of collagen XVII (KDColXVII-SCC-25, KDColXVII-HSC-3) or integrin β4 (KDβ4-SCC-25) were generated using virus-mediated RNA interference. Quantitative RT-PCR analysis of the remaining collagen XVII or integrin β4 expression showed efficient suppression varying between 78 to 91%. The results are averages of four parallel samples. The KD of collagen XVII or integrin β4 does not largely disturb the expression of each other or laminin γ2. (**b,c**) Immunoblotting with indicated antibodies and the quantification with ImageJ software confirmed that the expression of collagen XVII and integrin β4 is decreased in KD cells. GAPDH was used as a loading control. ColXVII = collagen XVII, ITGβ4 = integrin β4, Lamγ2 = laminin γ2. *p* Values are for independent sample *t*-test, *p < 0.05, ***p < 0.001. Full-length blots are presented in Supplementary Figure 2.

To address collagen XVII and integrin β4 function in SCC cells we generated SCC-25 and HSC-3 cells with either collagen XVII (KDColXVII-SCC-25 and KDColXVII-HSC-3) or integrin β4 (KDβ4-SCC-25) knockdown using virus-mediated RNA interference (RNAi). Analysis with qRT-PCR demonstrated that knockdown efficiency was 78% for KDColXVII-SCC-25, 85% for KDColXVII-HSC-3 and 91% for KDβ4-SCC-25 (Fig. 4a). Western blot analysis verified that the expression of collagen XVII and integrin β4 was significantly lower in the knockdown cells compared to the mock-transfected control cells (Fig. 4b,c).

**Figure 5:**
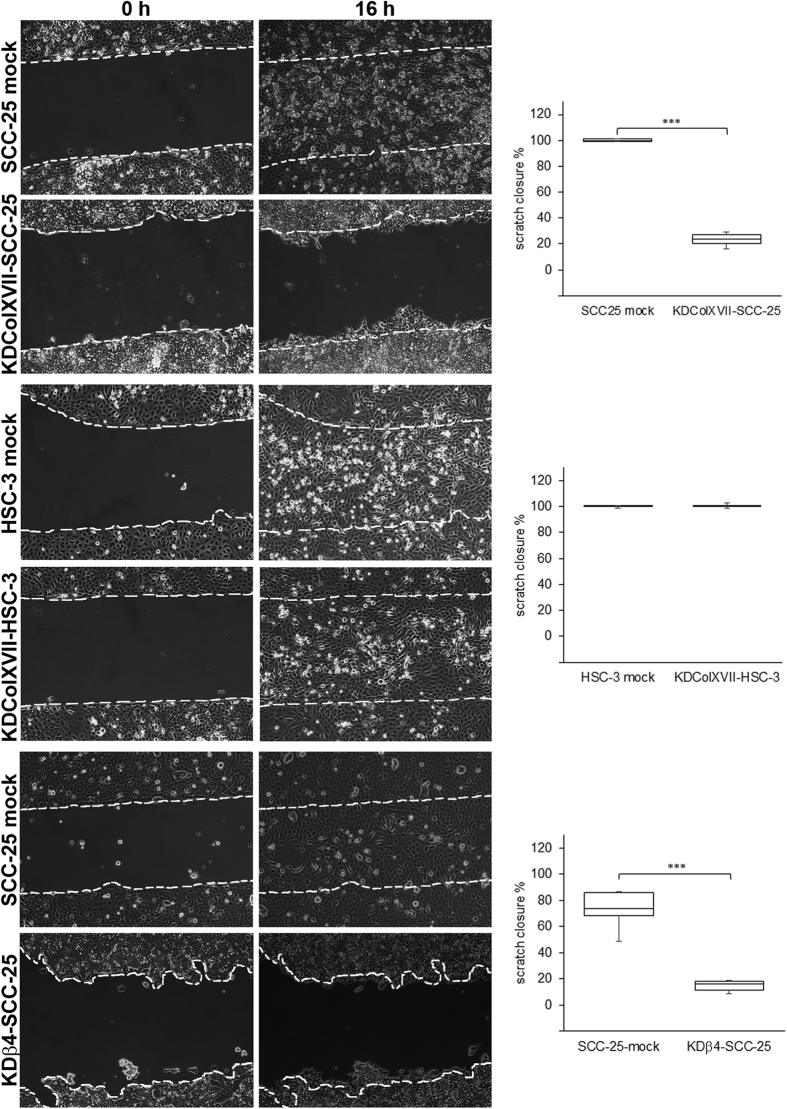
Collagen XVII and β4 integrin knockdown disturbs the migration of SCC-25 cells. In scratch assay both KDColXVII-SCC-25 and KDβ4-SCC-25 cells show highly disturbed migration compared to the control cells. In fact the movement of the KD-SCC-25 cells is stationary rotating and they are unable to close the wound whereas the control cells migrate rapidly and close the wound in 16 h time. KDColXVII-HSC-3 and control cells closed the wound in 16 hours. For quantification, the area of open scratch was measured using Image J. Each assay was performed in duplicates. *p* Values are for independent sample *t*-test, ***p < 0.001.

The migration of knockdown cell lines was analyzed using the scratch wound healing assay. The SCC-25 mock transfected cells started move across the scratched area rapidly within few hours, but the movement of both KDβ4-SCC-25 and KDColXVII-SCC-25 cells was stationary rotating. In sixteen hours, control cells closed the wound, but the migration of KDβ4-SCC-25 (*p* < 0.001) and KDColXVII-SCC-25 cells (*p* < 0.001) was significantly decreased (Fig. 5). Knockdown of collagen XVII had no effect to the migration of HSC-3 cells, since both KDColXVII-HSC-3 cells and mock-transfected control cells closed the wound in 16 hours (Fig. 5).

**Figure 6:**
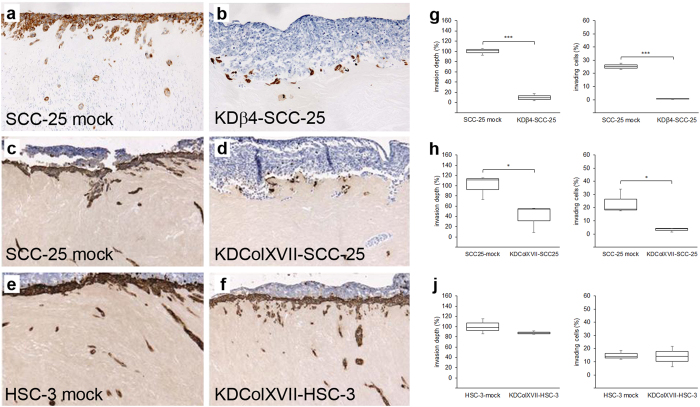
Collagen XVII and integrin β4 knockdown disables the invasion of the SCC-25 cells into the myoma tissue. The ability to invade was studied using organotypic myoma model. Same amount of SCC cells with collagen XVII or integrin β4 knockdown and their mock-transfected control cells were added on top of each myoma sample. Mock-transfected SCC-25 and HSC-3 cells invaded through the whole myoma section (**a,c,e**). KDβ4-SCC-25 cells (**b**) and KDColXVII-SCC-25 (**d**) did not invade to the myoma tissue at all, but KDColXVII-HSC-3 cells (**f**) invaded through the whole myoma section. For invasion analysis (**g–j**), myoma sections were immunostained with a monoclonal anti-human cytokeratin antibody. The areas of immunostained invading cells were calculated and the average invasion depth per microscopic field (the distance of the invading cell clusters from the lower surface of the non-invasive cell layer) as well as the area of invading cells was measured in each sample and compared to the total area of invading and non-invading cells. Each assay was performed in triplicates. *p* Values are for independent sample *t*-test, *p < 0.05,***p < 0.001.

The invasive properties of KDColXVII-SCC-25, KDβ4-SCC-25 and KDColXVII-HSC-3 cells were analyzed using an organotypic myoma model. Mock-transfected SCC-25 and HSC-3 cells invaded through the whole myoma section (Fig. 6a,c,e). SCC-25 cells lacking either integrin β4 or collagen XVII did not invade to the myoma tissue almost at all: the majority of KDβ4-SCC-25 and KDColXVII-SCC-25 cells remained at the surface of the myoma disc, and only a small proportion of them invaded (Fig. 6b,d,g,h). Both the invasion depth and area of invading cells of KDβ4-SCC-25 and KDColXVII-SCC-25 cells were significantly lower than those of mock-transfected control cells (Fig. 6g,h). KDColXVII-SCC-25 and KDβ4-SCC-25 cells, which were not able to invade and remained on the top of myoma disk, lost their keratin expression during the two week experiment, and thus lacked positivity in immunostaining with cytokeratin antibody (Fig. 6b,d). In contrast, majority of KDColXVII-HSC-3 cells invaded through the whole myoma disc and only a small amount of HSC-3 cells remained on the top of myoma disk (Fig. 6f,j).

## Discussion

Previous studies have reported that laminin 332, collagen XVII and integrin α6β4 are overexpressed in SCC^5^,^6^,^7^,^8^,^9^,^10^,^11^,^17^,^18^,^19^,^20^. Our study is the first to quantitate and compare the expression of all these three binding partners simultaneously through premalignant and carcinoma *in situ* lesions to cutaneous SCC tumors. Immunohistochemical staining of TMA samples demonstrated that the expression of laminin γ2 was highest in invasive SCC, but also increased in actinic keratosis and Bowen’s disease. This is similar to study by Hamasaki and co-workers who found that the expression of laminin γ2 is higher in SCC cases than in Bowen’s disease^9^. We found that the expression of collagen XVII is highly variable in actinic keratosis, Bowen’s disease and SCC samples. This differs from a previous TMA study, which showed that the expression of collagen XVII was more elevated in SCC samples than in actinic keratosis^20^. This discrepancy may arise from different scoring systems: We calculated the proportion of positive immunoreaction in epithelial cells relative to total epithelial cell area whereas in the earlier work the scoring was based on the number of immunopositive cell layers^20^. We also showed that the expression of collagen XVII and laminin γ2 are elevated in DMBA-TPA induced cutaneous papillomas and carcinomas of mice. This finding together with the results obtained from human samples demonstrates that collagen XVII and laminin γ2 seems to be involved in the pathogenesis of both ultraviolet light and chemically induced skin carcinogenesis.

The expression of integrin β4 has been reported to be higher in invasive SCC than in carcinoma *in situ* samples, but in this previous study, an antibody detecting the phosphorylated integrin β4 was used^27^. Immunostaining of phospho-β4 and laminin 332 did not co-localize, and the expression of collagen XVII was not analyzed^27^. Accordingly, we observed that the distribution pattern of laminin γ2 does not correspond with either integrin β4 or collagen XVII in SCC samples. However, the immunolocalization of collagen XVII and integrin β4 was very similar suggesting that they may interact with each other in SCC similarly to normal dermal-epidermal junction.

The mRNA levels for laminin γ2, integrin β4 and collagen XVII were increased in three out the four of analyzed SCC cell lines, and the amount of the collagen XVII shed ectodomain was increased in cell culture media of SCC cells. Previously the suppression of laminin γ2 by RNAi has been shown to inhibit significantly A431 SCC cell invasion^9^. This finding prompted us to investigate what is the effect of collagen XVII or integrin β4 virus-mediated RNAi knockdown on the behavior of SCC cells. We found that depletion of collagen XVII has no effect to the migration of the most aggressive HSC-3 cells, but, similar to keratinocytes, it disturbs the migration of less aggressive SCC-25 cells^13^,^15^,^28^. Current data on how the lack of collagen XVII changes keratinocyte migration are controversial: Keratinocytes without collagen XVII derived from human or mouse genetic models show increased, but undirected motility^13^,^29^, whereas keratinocytes with viral collagen XVII knockdown have reduced migratory ability^15^,^28^. We observed that KDColXVII-SCC-25 cells exhibit stationary rotation and markedly delayed migration in the scratch wound healing assay. Colon carcinoma cells with a lentiviral knockdown of collagen XVII were recently reported to display reduced migratory activity, reduced invasiveness and decreased wound closure activity^21^. Thus the effect of viral collagen XVII knockdown on cell migration is similar in keratinocytes, in SCC-25 cells as well as in colon carcinoma cells^15^,^21^,^28^. The depletion of collagen XVII and integrin β4 decreased the migration of SCC-25 cells in a similar manner. Our result is in line with the previous studies, which showed that the knockdown of integrin β4 reduces the migration of keratinocytes as well as malignant cells^30^,^31^,^32^,^33^.

The invasion properties of SCC cells with collagen XVII and integrin β4 knockdowns were analyzed using an organotypic assay, which is based on the use of human myoma tissue and mimics the tumor microenvironment better than collagen organotypic models^34^,^35^. The effect of collagen XVII or integrin β4 depletion to the invasion of less aggressive SCC-25 cells was similar and dramatic: Instead of invading to the myoma tissue as the control cells did, the majority of KDColXVII-SCC-25 and KDβ4-SCC-25 cells remained at the surface of the myoma disc. In contrast, the knockdown of collagen XVII had no effect to the migration or invasion of the most aggressive HSC-3 cells.

The evidence that the overexpression and signaling function of integrin β4 is significant in SCC and in several other malignancies is strong^11^. Our current data with suppression of SCC cells invasion may suggest a role also for collagen XVII in SCC tumorigenesis. The expression of collagen XVII is increased in melanoma and shows an association with invasive phenotype^36^. In line with our current results with collagen XVII-deficient SCC cells the overexpression of collagen XVII promoted the invasion of colon carcinoma cells^37^. Moreover, we and others have recently demonstrated that the expression of collagen XVII is increased in colorectal and lung cancers and correlates significantly with higher TNM stage, infiltrative growth pattern and metastasis^21^,^22^,^37^.

To conclude, immunohistochemical analysis of TMA samples demonstrated that the expression of laminin γ2 was highest in SCC, whereas the expression of collagen XVII and integrin β4 varied greatly in SCC and its precursors. In addition, the knockdown of collagen XVII and integrin β4 disturbed the migration and invasion of less aggressive SCC cells, but had no effect on the behavior of more aggressive SCC cells. The signaling of α6β4 integrin to regulate and promote the adhesion and migration of normal epithelial and cancer cells is mediated by the phosphorylation of the cytoplasmic tail of the integrin β4^10^,^11^. So far collagen XVII has been linked to the PI3K/Rac1 signaling in keratinocytes^13^, to the PP2A-STAT3 pathway in lung cancer stem cells^22^ and the FAK/AKT/GSK3β pathway in colon cancer stem cells^21^. Future studies exploring the effect of collagen XVII knockdown on various signaling pathways in SCC cells will help us to understand better the interaction between collagen XVII and integrin β4 in normal and malignant epithelial cells.

## Materials and Methods

### Patient tissue samples and tissue microarray

Tissue collection was performed and obtained from the archives of the Department of Pathology, Turku University Hospital. Tissue microarrays (TMAs) containing altogether 95 cases were included in the study (13 normal skin samples, 36 actinic keratosis samples, 30 Bowen’s disease samples and 16 SCC samples)^38^. The use of archival tissue specimens was approved by the Ethics Committee of the Hospital District of Southwest Finland, Turku, Finland. Before surgery, patients gave their informed consent, and the study was conducted according to the Declaration of Helsinki. To select optimal sample locations for tissue TMA the samples from surgical specimens were fixed in formalin solution, embedded in paraffin, and 5-μm sections were stained with hematoxylin-eosin. 1.5 mm punched cores (one core per case) from donor blocks were transferred to recipient TMA blocks^26^,^38^.

### Immunohistochemistry and immunoreactivity evaluation

Sections of 3.5 μm cut from TMA specimens were deparaffinized and rehydrated. After antigen retrieval and neutralizing endogenous peroxidase activity, the sections were incubated with the polyclonal NC16A antibody (1:8000)^39^, the monoclonal laminin γ2 (sc-28330, 1:2000) and the polyclonal integrin β4 (sc-9090, 1:2000) (Santa Cruz Biotechnologies Inc., Santa Cruz, CA, USA). The EnVision^TM^ system (Dako Denmark A/S, Glostrup, Denmark) was used for detection. The histological images were captured with an Olympus DP25 camera (Olympus, Center Valley, PA, USA) attached to a Nikon Eclipse E600 microscope (Nikon, Tokyo, Japan) using x20 objective.

Computer-assisted immunoreactivity evaluation was done using ImageJ (NIH, Bethesda, MD, USA), a freeware image analysis software, using a method described and validated earlier^40^. Representative areas of the TMA punches were selected and the macro was used to calculate the proportion of positive immunoreaction in epithelial cells relative to total epithelial cell area in the selected region of interest representing normal skin, actinic keratosis, Bowen’s disease or SCC. TMA punches with insufficient tissue or tumor were excluded from the study.

### Chemical skin carcinogenesis

Chemical skin carcinogenesis of wild type mice with FVB/N background was induced using the multistage DMBA-TPA protocol as described earlier^41^. The skin tumor samples were evaluated in a blinded manner on the basis of hematoxylin-eosin stained sections. Immunohistochemical staining was performed using polyclonal NC14A (1:5000^42^) and monoclonal laminin γ2 (sc-28330, 1:2000, Santa Cruz Biotechnologies, Inc., Santa Cruz, CA, USA) antibodies. Detection was done using Dako EnVision Kit.

### Cell cultures and Western blotting

HaCaT cells (a generous gift from Dr. N. Fusenig, German Cancer Research Center, Heidelberg, Germany) were cultured in serum-free keratinocyte medium supplemented with recombinant epidermal growth factor and bovine pituitary extract (Invitrogen, Paisley, UK). Human oral tongue SCC lines SCC-15, SCC-25 (ATCC cultures, Teddington, UK) and HSC-3 (JRCB 0623, Japan Health Science Research Resources Bank, Osaka, Japan) were cultured in Dulbecco’s Modified Eagle Medium (DMEM) F-12 (Life Technologies, Grand Island, NY, USA) supplemented with 10% fetal calf serum, 2 mM L-glutamine, 15 mM HEPES, 400 ng/ml hydrocortisone (Sigma-Aldrich St. Louis, MO, USA) and 5 U/ml penicillin/streptomycin (Life Technologies). Sodium Bicarbonate (Invitrogen) and 250 ng/ml fungizone amphotericin B (Life Technologies) were added to SCC-15 and SCC-25 cells. Human primary SCC cell line UT-SCC-105 was established from surgically removed SCC of skin and cultured as described^26^.

For immunoblotting cultured cells and media were processed separately. Transmembrane proteins were extracted from the cells as described earlier^39^. Briefly, after washes the cell layer was incubated on ice with extraction buffer containing both detergent (1% Nonidet P-40) and protease inhibitors (7 μg/ml antipain, 7 μg/ml leupeptin, 14 μg/ml pepstatin, 14 μg/ml chymostatin, all from Sigma-Aldrich), 1 mM Pefablock (Merck, Darmstadt, Germany) and 10 μl/ml N-ethylmaleimide (NEM) (Sigma-Aldrich). The lysate was scraped from the culture dish, centrifuged to remove the cellular debris and the supernatant was used in analyses. SDS-PAGE and immunoblotting with the polyclonal anti-human collagen XVII antibody NC16A (1:1000)^39^ and the polyclonal anti-integrin β4 (sc-9090, 1:1000, Santa Cruz Biotechnologies Inc.) and anti-GAPDH (sc-25778, 1:500, Santa Cruz Biotechnologies Inc.) were performed using standard techniques. Peroxidase conjugated anti-rabbit IgG (Sigma-Aldrich) as a secondary antibody, ECL Prime chemiluminescence substrates (GE Healthcare, Buckinghamshire, UK) and LAS-3000 Image analyzer system (Fujifilm, Tokyo, Japan) were used for the visualization of specific protein bands. Specific protein bands were quantified with ImageJ software (NIH, Bethesda, MD, USA).

### RNA isolation and quantitative RT-PCR

Total RNA was isolated from cultured cells using QIAamp RNA Blood Mini Kit (Qiagen, Crawley, UK). The mRNA levels were measured by quantitative RT-PCR (qRT-PCR) analysis using SYBR Green Supermix (Bio-Rad, Hercules, CA, USA) in a final volume of 25 μl by iQ5 multicolour real-time PCR equipment (Bio-Rad). cDNAs were synthesized by standard methods (Thermo Fisher Scientific, Waltham, MA, USA) and the mRNA levels were analyzed by qRT-PCR using following primer pairs: human collagen XVII (NM_000494) forward 5′-ATGGGGACAGCATGGATAGA-3′ and reverse 5′-CTGGACTTCCCATGTCACCT-3′, laminin γ2 forward 5′- GTCACTGGAGAACGCTGTGA-3′ and reverse 5′ AGACCCATTTCGTTGGACAG-3′, integrin β4 5′- TGGAAGTACTGTGCCTGCTG-3′ and reverse 5′ TGCATGTTGTTGGTGACCTT-3′ and beta-actin 5′-AGAGCTACGAGCTGCCTGAC-3′and reverse 5′-AGCACTGTGTTGGCGTACAG-3′. The relative change in collagen XVII, laminin γ2 and integrin β4 mRNA expressions were calculated by Livak’s 2^−∆∆C^_T_ method^43^.

### Collagen XVII and β4 integrin knockdown

The generation of SCC-25 and HSC-3 with collagen XVII knockdown by retrovirus-mediated RNAi was performed as described in more detail previously^44^. Cells were infected with retroviruses encoding short hairpin RNAs (shRNAs) designed for the depletion of the specific target genes (constructed in the vector RVH1-puro). The retroviral knockdown strains with stable genomic integration of the shRNA expression cassettes were selected in the presence of puromycin (2 ng/ml) (EMD Millipore, Billerica, MA, USA) in cell culture medium. β4 integrin knockdown was generated using MISSION shRNA lentivirus (Sigma, München, Germany) according to the manufacturer’s protocols. The knockdown efficiency (the percentage reduction of the target gene mRNAs in comparison to the level in control cells) was determined by qRT-PCR. Three different collagen XVII and five different β4 integrin constructs were tested and the one with most efficient and most stable knockdown was chosen for migration and invasion assays.

### Cell migration and invasion analysis

The migration assay was performed using Ibidi Culture-Inserts (Ibidi, Martinsfried, Germany) on glass-bottom 6 well plates (MatTek Corporation, Ashland, MA, USA). 14,000 cells were added to insert chambers and 380,000 cells in every well. After reaching the confluence (48 h) the inserts were removed to generate a wound and medium was changed. The imaging was performed using Olympus Cell^P live-cell/timelapse imaging system. Images were taken every 20 minutes until the wound was closed in control cells. Open scratch area after 16 h was measured from time-lapse images with ImageJ software (NIH, Bethesda, MD, USA) and compared to scratch area in the beginning of the experiment. Each assay was performed in duplicates and time-lapse imaging was performed for 4–6 areas per sample.

Invasive properties of SCC-25 and HSC-3 cells with collagen XVII or β4 integrin knockdown and control cells transfected with an empty vector were analyzed using an organotypic myoma model according to method described earlier^35^. In brief, myoma disks were first equilibrated in media at room temperature for 1 hour and then placed into Transwell inserts (diameter 6.5 mm, Corning, Inc., Corning, NY, USA). Next, 7 × 10^5^ cells in 50 μl of media were added on top of each myoma disk and allowed to attach overnight. The myoma disks were then removed from the Transwell inserts and transferred onto uncoated nylon disks resting on curved steel grids (3 × 12 × 15 mm) in 12-well plates with sufficient volume of media (1 ml). The myoma organotypic cultures were maintained for 11–14 days, and the media was changed every 72 h. Each assay was performed in triplicates. The specimens were fixed in formalin overnight, dehydrated, bisected, and embedded in paraffin. Finally, sections (6 μm) were deparaffinized and stained with monoclonal mouse anti-human cytokeratin (Dako Denmark A/S, Glostrup, Denmark). The areas of immunostained invading cells were calculated, and the average invasion depth per microscopic field (the distance of the invading cell clusters from the lower surface of the non-invasive cell layer) as well as the area of invading cells was measured in each sample and related to total surface area of non-invading and invading cells. Measurements were performed with ImageJ software.

### Statistical analysis

The statistical analyses were conducted using IBM SPSS Statistics for Windows, version 22.0 (IBM Corp., Armonk, NY, USA). The independent sample *t*-test was used to analyze the statistical significances in immunoblotting, scratch wound healing and organotypic myoma assays. The statistical significances of the associations of the amount of positive immunoreaction in epithelial cells with different tumor types were analyzed by Kruskal-Wallis test (comparing three or more classes) or Mann-Whitney *U* test (comparing two classes). Spearman rank correlation coefficients (ρ) were used to study the correlation between different immunohistochemical markers. A two-tailed *p* value < 0.05 was considered statistically significant.

## Additional Information

**How to cite this article**: Moilanen, J. M. *et al*. Significant Role of Collagen XVII And Integrin β4 In Migration and Invasion of The Less Aggressive Squamous Cell Carcinoma Cells. *Sci. Rep.* **7**, 45057; doi: 10.1038/srep45057 (2017).

**Publisher's note:** Springer Nature remains neutral with regard to jurisdictional claims in published maps and institutional affiliations.

## Supplementary Material

Supplementary Dataset 1
