# Supplementary material for: Significant Role of Collagen XVII And Integrin β4 in Migration and Invasion of The Less Aggressive Squamous Cell Carcinoma Cells
Source: Sci Rep. 2017 Mar 22;7:45057. doi: 10.1038/srep45057 (PMC5361192; doi:10.1038/srep45057)
Supplement: Supplementary Dataset 1 [file srep45057-s1.doc]

**SIGNIFICANT ROLE OF COLLAGEN XVII AND INTEGRIN β4 IN MIGRATION AND INVASION OF THE LESS AGGRESSIVE SQUAMOUS CELL CARCINOMA CELLS**

Jyri M. Moilanen^1^, Stefanie Löffek^2^, Nina Kokkonen^1^, Sirpa Salo^3^, Juha P. Väyrynen^4^, Tiina Hurskainen^1^, Aki Manninen^3^, Pilvi Riihilä^5^, Ritva Heljasvaara^3^, Claus-Werner Franzke^6^, Veli-Matti Kähäri^5^,Tuula Salo^7^, Markus J. Mäkinen^4^, Kaisa Tasanen^1^*

**SUPPLEMENTARY MATERIAL**

**
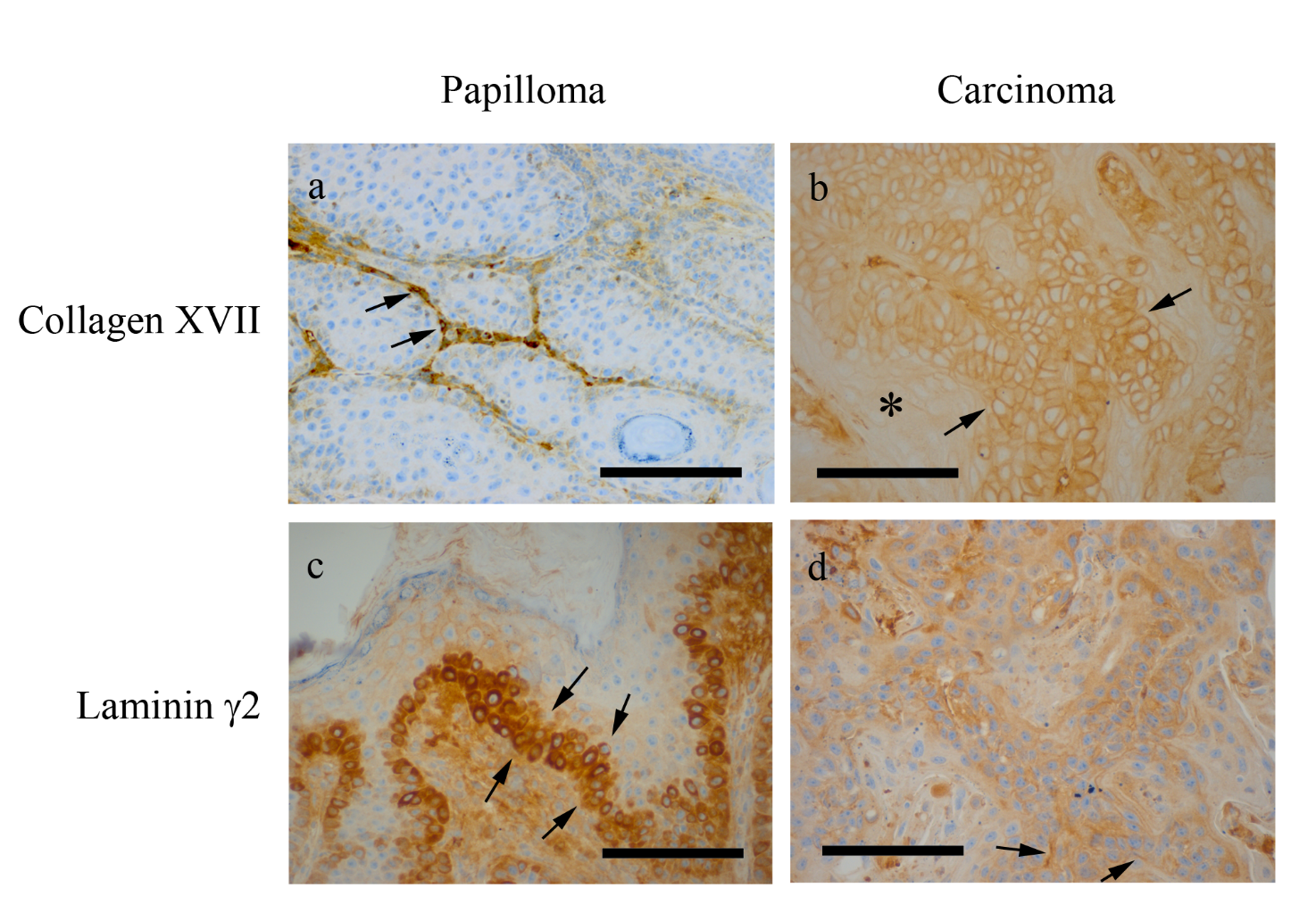
**

**Figure S1***.* Collagen XVII and laminin γ2 are up-regulated in chemically induced skin tumors of mice. The expression of collagen XVII and laminin-332 in the DMBA-TPA carcinogen-induced murine skin tumors. Tumors were induced in the FVB/N mouse skin using the multistage DMBA-TPA protocol and their growth was monitored. In benign papillomas, both collagen XVII (a) and laminin γ2 (c) are observed in basal cells (arrows). SCC shows mostly membranous immunoreaction against collagen XVII (b) in proliferating cells (arrows), whereas differentiated cells show weaker reaction or are negative (asterisk). Similarly, SCC shows variable reaction intensity for laminin γ2 (d), staining being both cytoplasmic and membranous (arrows). Original magnification 200x. Scale bar 100 μm.


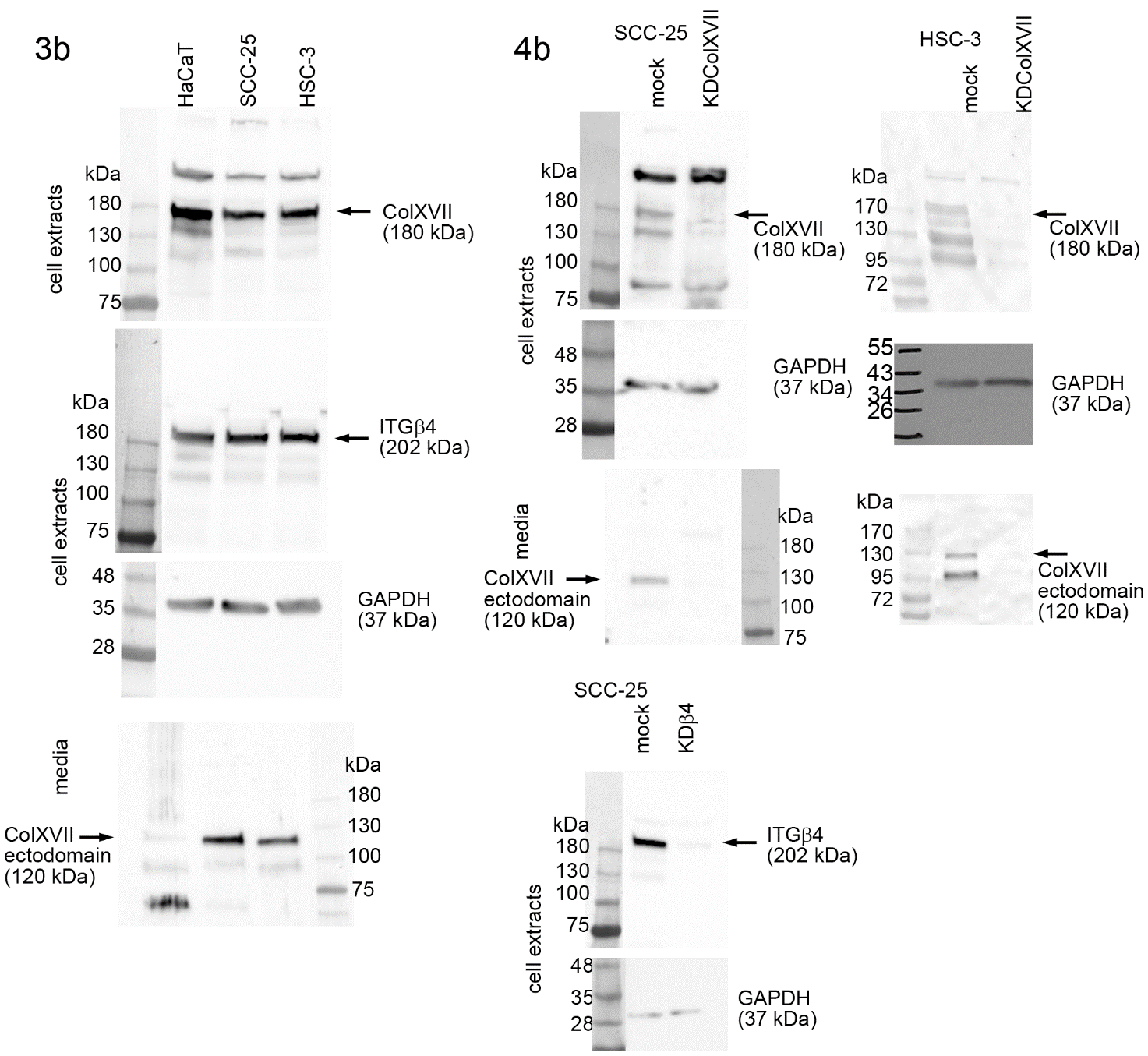


**Figure S2**. Original full length blots.
